# Revealing the Relational Mechanisms of Research for Development Through Social Network Analysis

**DOI:** 10.1057/s41287-023-00576-y

**Published:** 2023-01-25

**Authors:** Marina Apgar, Guillaume Fournie, Barbara Haesler, Grace Lyn Higdon, Leah Kenny, Annalena Oppel, Evelyn Pauls, Matthew Smith, Mieke Snijder, Daan Vink, Mazeda Hossain

**Affiliations:** 1 Institute of Development Studies, University of Sussex, Library Road, Falmer, Brighton, BN1 9RE East Sussex UK; 2 Royal Veterinary College, 4 Royal College St, London, NW1 0TU UK; 3 London School of Economics, Houghton St, London, WC2A 2AE UK; 4 Edinburgh Napier University, Sighthill Campus, Sighthill Court, Edinburgh, EH11 4BN UK

**Keywords:** Social network analysis, Collaboration, Relational, Evaluation, Learning, Research for Development

## Abstract

Achieving impact through research for development programmes (R4D) requires engagement with diverse stakeholders across the research, development and policy divides. Understanding how such programmes support the emergence of outcomes, therefore, requires a focus on the relational aspects of engagement and collaboration. Increasingly, evaluation of large research collaborations is employing social network analysis (SNA), making use of its relational view of causation. In this paper, we use three applications of SNA within similar large R4D programmes, through our work within evaluation of three Interidsiplinary Hubs of the Global Challenges Research Fund, to explore its potential as an evaluation method. Our comparative analysis shows that SNA can uncover the structural dimensions of interactions within R4D programmes and enable learning about how networks evolve through time. We reflect on common challenges across the cases including navigating different forms of bias that result from incomplete network data, multiple interpretations across scales, and the challenges of making causal inference and related ethical dilemmas. We conclude with lessons on the methodological and operational dimensions of using SNA within monitoring, evaluation and learning (MEL) systems that aim to support both learning and accountability.

**Supplementary Information:**

The online version contains supplementary material available at 10.1057/s41287-023-00576-y.

## Résumé

Pour que les programmes de recherche pour le développement (R4D ou Research for Developmement en anglais) aient un impact, il faut un engagement entre diverses parties prenantes dans les domaines de la recherche, du développement et des politiques. Il est nécessaire de se concentrer sur les aspects relationnels de l'engagement et de la collaboration si l’on souhaite comprendre la façon dont ce type de programme permet l’émergence de résultats. L'évaluation des grands consortia de recherche utilise de plus en plus fréquemment l'analyse des réseaux sociaux (SNA ou social network analysis en anglais) en appliquant sa vision relationnelle de la causalité. Dans cet article, en vue d'explorer son potentiel en tant que méthode d'évaluation, nous utilisons trois applications d’analyse des réseaux sociaux au sein de grands programmes R4D similaires dans le cadre de notre travail d'évaluation de trois pôles interdisciplinaires du Fonds de recherche sur les défis mondiaux. Notre analyse comparative montre que l’analyse des réseaux sociaux peut révéler les dimensions structurelles des interactions au sein de ces programmes et permettre d'apprendre comment les réseaux évoluent dans le temps. Nous menons une réflexion quant aux défis communs qui émanent de ces cas, y compris la gestion de différentes formes de biais qui résultent de données de réseau incomplètes, de multiples interprétations sur des échelles différentes et les défis liés au fait d’établir une inférence causale et les dilemmes éthiques connexes. Nous concluons par des leçons sur les dimensions méthodologiques et opérationnelles de l'utilisation de l’analyse des réseaux sociaux dans les systèmes de suivi, d'évaluation et d'apprentissage (SEA) qui visent à soutenir à la fois l'apprentissage et la redevabilité.

## Introduction

Research for development programmes (R4D) aim to put research at the service of solving intractable development challenges, and often have a focus on improving livelihoods of marginalised or excluded populations. Achieving development outcomes for these populations requires engagement with diverse stakeholders across the research, development and policy divides. Relationships between partners within R4D networks are central mechanisms for shaping activities as well as engagement and impact strategies through collaboration (Temple et al. 2018). Outcomes emerge from these interactions, leading to uncertainty in their pathways to impact (Jacobi et al. 2020; Maru et al. 2018; Thornton et al. 2017). Understanding if and how R4D programmes support the emergence of outcomes, therefore, requires a focus on the relational aspects of engagement and collaboration.

In the case of the Global Challenges Research Fund of the UK the funder, UKRI, set the ambition for the portfolio of R4D programmes well beyond the delivery of world class research, and included partnerships between the UK and the Global South as a desired outcome, requiring strong networks to be built between diverse stakeholders (Barr et al. 2019).^1^ The scale of the GCRF portfolio (initially proposed at £1.5billion) embracing R4D programme as network building initiatives, emphasising collaboration and learning across the research and development sectors, created an unprecedented opportunity to deepen understanding of the relational mechanisms that contribute to achieving outcomes and impact.

In this paper, we explore methodologies from within GCRF programmes used in evaluating them as network building initiatives. We focus on the use of social network analysis (SNA) as one tool in an R4D methodological repertoire. Although SNA alone is not sufficient to fully understand the contribution of these complex programmes to development outcomes and impact, our comparative analysis shows that it can uncover the structural dimensions of interactions within large R4D programmes and enable learning about how networks evolve through time. We reflect on common challenges across the cases including navigating different forms of bias that result from incomplete network data, multiple interpretations across scales, the challenges of making causal inference and related ethical dilemmas. We conclude with lessons on the methodological and operational dimensions of using SNA within monitoring, evaluation and learning (MEL) systems with dual aims of supporting both learning and accountability.

## SNA Within Complexity-Aware Evaluation

The uncertainty of impact pathways in R4D programmes, and the need to centre the network of social actors and their interactions throughout implementation call for evaluation designs that focus on explaining how change is unfolding in real time, often referred to as complexity-aware (Bamberger et al. 2015; Douthwaite and Hoffecker 2017; Gates and Fils‐Aime 2021; Patton 2010). These designs respond to understanding development programmes, policies and interventions as operating under conditions of complexity, requiring multiple strategies and engagement with diverse actors within systems. Programme outcomes emerge from interactions between the parts (relationships between actors) rather than from what individual parts achieve alone (Hargreaves 2021; Walton 2016). This is even more evident when programmes are working in conflict-affected contexts which are highly dynamic. Such programming requires non-linear evaluation designs to capture emergent outcomes through the interactions, as well as understanding achievement of intended outcomes, and emphasize iterative learning as change happens (Apgar et al. 2020). These new approaches to evaluation offer opportunities for focussing on the interactions between actors in an R4D network. Within these broad designs, there is a need to zoom into the structural dimensions of collaboration in order to then explore causal relationships between networking and intended outcomes, along impact pathways.

SNA is a recognised interdisciplinary methodological field within social science research, building on its sociological and mathematical (graph theory) roots (Freeman 2000). One of the central offerings of SNA is its relational view of causation, as Marin and Wellman (2011, p. 13) describe it “social network analysts argue that causation is not located in the individual, but in the social structure”. Using SNA as a method allows for intuitive visualisations of relationships as well as tangible measures of “network quality” (Davies 2009). Analytical approaches for SNA are diversifying (including quantitative, qualitative, and mixed strategies), and combining structural and relational approaches to causation is leading to greater exploration of its use for evaluation. As a recent scoping review of the use of SNA in evaluation shows, there is a steady increase in its application since the turn of the century (Popelier 2018) increasing its potential to support evaluation of complex systems. A number of applications are relevant to the R4D programming context (e.g. Aboelela et al. n.d.; Drew et al. 2011; Haines et al. 2011; Honeycutt and Strong 2012) and highlight both opportunities and challenges. In this paper, we add to this nascent field through comparative analysis of three experiences of SNA in the context of large R4D programmes.

## Methodology

We use a case study methodology (Yin 1989) to learn within and across three applications of SNA in similar large interdisciplinary collaborations funded as Interdisciplinary Hubs by UKRI under the GCRF—we will refer to these R4D programme as ‘Hubs’. They have sufficient similarity in scale and approach to evaluation to support cross-case analysis, while each application is necessarily bespoke to its programme context and needs. Table 1 summarises each of the cases, showing that evaluation and research questions that drove the use of SNA in each Hub differ slightly, and consequently, the design of the data collection tools and analytical strategies also differ (justification for each analytical strategy can be found in the Online Technical Appendix).

**Table 1:** Three cases of use of SNA in GCRF funded interdisciplinary research Hubs

Hub	Timeframe/frequency	Node attributes	Relational attributes	Evaluation and learning questions
One Health Poultry Hub	Before hub inception (retrospective), 12 months since inception, annually thereafter	Gender, career stage, country, discipline	Specific categories of interactions (e.g. research, outreach, administration)	To assess the way in which collaborations are being shaped among Hub members and investigate whether the emerging network is showing changes in terms of centralisation and hierarchy. Part of evaluation that aims to investigate how key pillars of capability building, namely collaboration, learning and sharing, are evolving in the Hub
Gender, Justice and Security Hub	Before Hub inception (retrospective), 16 months since inception (June 2020)	Geography, career stage, stream (theme), type of institution	Strength of connections, origin of connections (non-Hub, Hub, specific Hub activity)	Part of a broader research project to understand the relationship between the Hub and a larger network of Women, Peace and Security practitioners. Part of evaluation of Hub Impact 1: “New knowledge and advocacy networks amplify the voices of women and marginalised groups to catalyse change across issues and sites”
Tomorrow’s Cities Hub	After 2 years of implementation and subsequently after major Huub events, and as part of annual reviews	Discipline, geography (city affiliation), gender type of institution, career stage	Strength of interactions (capturing both formal and informal)	Component of theory-based evaluation. Initial stages responding to the evaluation question: How is the Hub building a network of interdisciplinary and engaged researchers focussed on urban disaster risk reduction? In later stages evaluating the contribution of the Hub to shifts in urban risk governance (main outcome and impact areas for the Hub)

The Hubs experienced two major disruptions in the early phases of implementation that influenced both the network formation processes and relatedly the application of the SNA method; (i) the COVID-19 pandemic required all Hubs to adapt to online collaboration and many network forming activities were no longer possible, and (ii) an unexpected and significant reduction in funding (due to a reduction in overall UK government funding for ODA) led to loss of staff and reduced scope of monitoring activities for a 12-month period. Our focus in this paper, therefore, is necessarily on the initial phases of work. All three cases include a baseline application of SNA with the shared goal of assessing the way in which collaborations were shaped through the early phases of implementation, and where possible, how this was influenced by the disruptions experienced.

We, the co-authors, are the designers and implementers of the SNA within the Hubs, involved as researchers, MEL specialists, data analysts and programme managers. The within-case analysis was carried out by each programme team independently, following its own strategy, and focussed on what the SNA revealed about the particular evaluation and learning goals. We were not external researchers using SNA to understand the programmes but active users of the method as a mechanism for programmatic learning through our positions within each Hub. The diversity of roles we played has enabled analysis across methodological, operational and strategic layers of use of SNA as an evaluation method.

## Learning from Use of SNA

In this section, we summarise the application of SNA in each case and present the findings from within-case analysis. Full technical details of the SNA applications in each Hub are presented in the Online Technical Appendix and illustrate that analytical strategies were specific to each case. In all cases, we reflect on whether the SNA findings primarily displayed aspects of project design (controlled) or social collaboration that occurs within the project (uncontrolled) in the early phases of programme implementation.

### One Health Poultry Hub

The One Health Poultry Hub (OHPH) addresses zoonotic disease risks associated with poultry intensification, with a geographic focus on Bangladesh, India, Sri Lanka and Viet Nam. To address this challenge and ensure the safe and sustainable production of poultry, it aims to promote interdisciplinary and cross-sectoral dialogue within a One Health environment. Indeed, given the cross-cutting nature of these issues, strengthening interdisciplinary research capability and competencies, collaboration and knowledge exchange are core activities. Assessment and monitoring of such attributes over the OHPH’s lifetime form part of the Hub’s MEL framework. Given the programme design we expected that (i) connections between study countries would be mainly mediated by a small number of UK partners in the early network, and (ii) the network structure would then become less centralised in the later study periods, with more direct connections between study country partners. A key principle driving the evaluation and so the demand by the UK management team was to produce learning to feed adaptive programme management and encourage decentralised network growth.

In this context, we applied SNA methods to investigate the evolution of the OHPH network, a dynamic partnership network consisting of approximately 120 named researchers from 27 institutions in 10 countries. Specific objectives were (i) to assess the way in which collaborations were being shaped among its members during the course of the project; (ii) to characterise the extent to which the emerging network is dynamically changing across countries and research areas; and (iii) to investigate characteristics in the development of the OHPH network associated with factors such as career stage, scientific discipline and gender.

#### Methods

The SNA was conducted using data from two bespoke online surveys (see full technical details in the Online Technical Appendix). The first was carried out in March 2020, one year after the Hub’s launch, and the second in February 2021. All co-investigators and researchers engaged with the Hub, contracted research staff, postgraduate students and managerial staff were invited to respond (120 named researchers). Respondents were asked to consider their collaborations and activities with all other Hub members over three periods: P0 (before the Hub’s inception), P1 (during the first year of the Hub) and P2 (during the second year of the Hub). In addition, respondents were asked to indicate their primary scientific discipline or area of expertise, their primary role in the Hub, gender, and age category.

#### Findings

While some respondents filled the survey for all three periods, others provided information for only one or two. For each period, we, thus, considered two sets of nodes: all respondents who responded within each period (period-specific networks), and respondents who completed all three questionnaires (cohort networks) for which changes in connection patterns over time among the same set of nodes can be assessed. The comparison of cohort and period-specific networks allows us to assess whether analytical results are affected by the composition of our sampled networks (i.e. selection bias). All networks were undirected: if at least one respondent reported a collaboration with another respondent, an edge was constructed between them. The size of the period-specific networks ranged from 58 to 81 nodes (35 to 45% of Hub partners). The cohort networks had 37 nodes (see Online Technical Appendix for details). About two thirds of respondents were from the study countries, and almost all others were based in the UK. Most respondents were male, biological scientists, and at mid to late career stage.

**Fig. 1:**
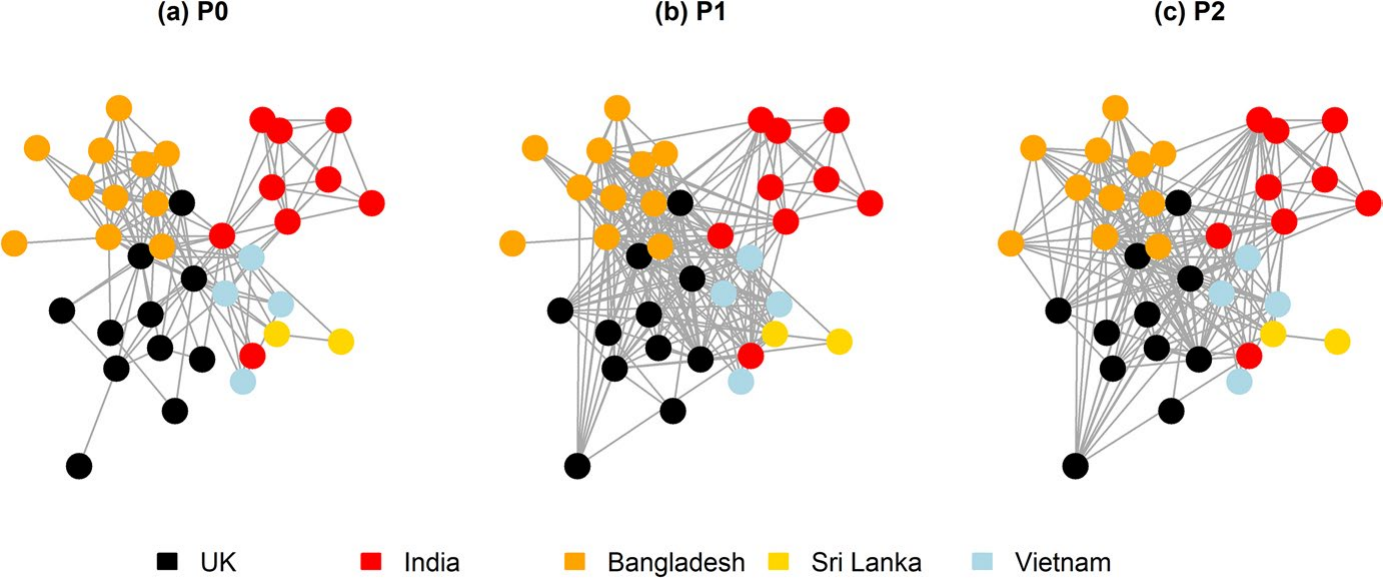
Network diagrams showing the OHPH cohort networks (network of those who responded to all three time periods). Nodes are coloured according to the country in which they were based

Each period-specific and cohort network showed a high small-world index which is indicative of high clustering and short path lengths between nodes (Humphries and Gurney 2008) See Fig. 1.  From P0 to P1, the proportion of connected dyads increased as the OHPH’s project activities started. This increase in connectedness was distributed among partners, reducing the extent to which a small number of actors acted as mediators between most others. This small-world structure, and the evolution towards a reduction in centralisation would be expected to promote the diffusion of information and knowledge, and their equitable access by Hub members. Several face-to-face meetings were organised during P0 and P1, including a whole-Hub conference. Such meetings enabled partners from all disciplines and partner countries to meet and collaborate directly. This was likely a major driver in increasing the network connectedness and reducing its centralisation when compared to the pre-inception period. However, the COVID -19 pandemic effectively eliminated all such opportunities from the OHPH’s second year (which commenced in March 2020). Similar to other GCRF interdisciplinary Hubs, all OHPH-wide events, regular project coordination meetings, meetings of working groups leading the design and implementation of research, impact and learning activities, ad-hoc workshops, conferences, early career researcher group meetings and other opportunities for interaction were migrated to online platforms. Possibly as a result of this, we found that the network’s connectedness decreased from P1 to P2 as well as it becoming more centralised—that is, more connections were mediated by a small number of highly-connected nodes. This pattern was observed in the period-specific as well as cohort networks.

We assessed whether the centrality of a node was associated with its attributes (country, discipline, career stage, gender) using multivariable permutation-based linear models (as in Delabouglise et al. 2017). We considered two centrality measures: degree (the number of other nodes with which a node was connected) and betweenness (the extent to which a node lay on the shortest path between two others). For the period-specific network at P2, there was weak evidence that the average degree could be lower for study countries than for UK partners, and for women than for men. Active participation in P2 online events varied depending on internet access, bandwidth and quality. Not all participants had access to the required IT hardware when working from home, and it was apparent that online formats made participation more challenging for partners for whom English is a second language. These associations were not, however, observed on the cohort network. Betweenness was not associated with any abovementioned node-level attributes.

We assessed the possible influence of individual respondent factors (country, discipline, career stage, gender) on the occurrence of edges between any two nodes. We found that, for all periods and network types, the likelihood of a connection increased if two partners were from the same country, but decreased if they were both from different study countries. By the second year of the Hub’s operation (P2), all UK and 82% of study country partners were engaged in connections between the UK and study countries, whereas connections across study countries only involved 47% of study country partners. The connectedness was higher among social scientists, mid and late career stage and male partners in the period-specific network. These associations were not, however, found in the cohort-specific networks. The design of the OHPH’s research programme, which was initiated in the second year, was to an extent replicated across the countries in which it was implemented (to enable standardisation and comparability of outcomes), but required modifications for each of the study sites to enable specific research questions to be addressed, as well as incorporating local differences. This required central coordination by the core research and management team (based primarily in the UK) as well as intensive collaboration within the site teams. It is likely that this further contributed to centralisation and compartmentalisation of the network over the P2 period. Moreover, online meetings might have made it more difficult for participants who may perceive themselves as being lower in the hierarchy of their institution (e.g. early career researchers) to express opinions or share knowledge.

#### Reflections on Contributions of SNA for MEL

While SNA has been useful to visualise and assess changes in connectivity and centrality of the OHPH’s network, care should be taken not to overinterpret these results. A major limitation in this analysis is the likely selection bias. Only a small proportion of the total hub partners (between 35 and 45%) took part in the study. The composition of the respondent groups was likely to be non-representative in the two surveys, as well as varying between the two surveys. Hence, depending on the factors which affected participation, some of the results may only be valid for the group of respondents and not the entire collaborative network, as suggested by the discrepancies in some analytical results between the cohort and period-specific networks.

Nevertheless, the implementation of SNA using a repeated annual survey was found to be helpful to characterise the changing nature of the OHPH network over its lifetime, and provide insights into the dynamic processes and factors (some of which are external) which influence this. It allowed the Hub to assess whether the network structure was evolving towards the emergence of desirable characteristics, such as a reduced UK-focus centralisation, increased interactions across study countries and disciplines and reduced influence of one’s career stage and gender on node centrality. Although the limitations mentioned previously imply that the scope for SNA alone to explore or quantify such nuanced or complex issues is limited, it should be considered as a tool that can be applied alongside output-based indicator measurement approaches.

### Gender Justice and Security Hub

Gendered political, economic and social injustices shape the outbreak and dynamics of conflict; war itself involves violence against women and girls as well as violations of other human rights; and redress from the gendered harms of war is intimately connected to the establishment of a lasting and just peace. In short, gender is a fundamental web of social relations through which justice and security are mediated. The Gender, Justice and Security Hub (GJSH) responds to this challenge through bringing together 121 members across 42 partner organisations from a variety of disciplinary perspectives, skill sets, career stages and geographic locations—with Afghanistan, Colombia, Kurdistan-Iraq, Lebanon, Sierra Leone, Sri Lanka and Uganda as focus countries and 17 more in which project work takes place. It aims to achieve the creation and growth of a network of academics, activists, practitioners and policymakers to advance progress towards gender justice and inclusive security in conflict-affected societies.

A central component of the Hub’s MEL plan was tracking whether and how network collaborations and connections develop and change over time.. A quantitative social network analysis was used to understand how collaborations were being shaped during the course of the project so that they could produce learning to inform programme adaptation. The study was to be applied at every year of the Hub’s lifetime (2020–2024); however, due to the ODA funding cuts, we only report on the first round of the survey in this paper. The objectives of the SNA were (i) to map the Hub network in order to visualise its overall structure, including density and strength of connections between members; (ii) to document how network connections change in number and strength over time; (iii) to identify patterns of connections within the network by attributes (by stream, career stage, geography, etc.) and (iv) to facilitate introductions among Hub members to improve the number and depth of collaborative relationships within the Hub. The expectation was that the Hub would be a fairly centralised network in its early stages given that the setting up of the Hub-level structure was run by a central Management Impact Communications Administration (MICA) team based in the UK and the Executive Group, consisting of 12 Co-Directors leading six research streams.^2^ The Hub activities were designed to strengthen and expand connections over time between Hub members who are not in the core group during the initial setting up period, leading to a less centralised network structure.

#### Methods

Data were collected between June and December 2020. Our sampling framework included all individuals associated with the GJSH at the time of the study (121 individuals). This included individuals directly or indirectly involved with the Hub research and advocacy-related activities (e.g. administrative staff, research partners, management, activists and Hub Champions). Nearly half (45%) of GJSH members completed the full survey. (See further methodological details in the Online Technical Appendix).

#### Findings

**Fig. 2:**
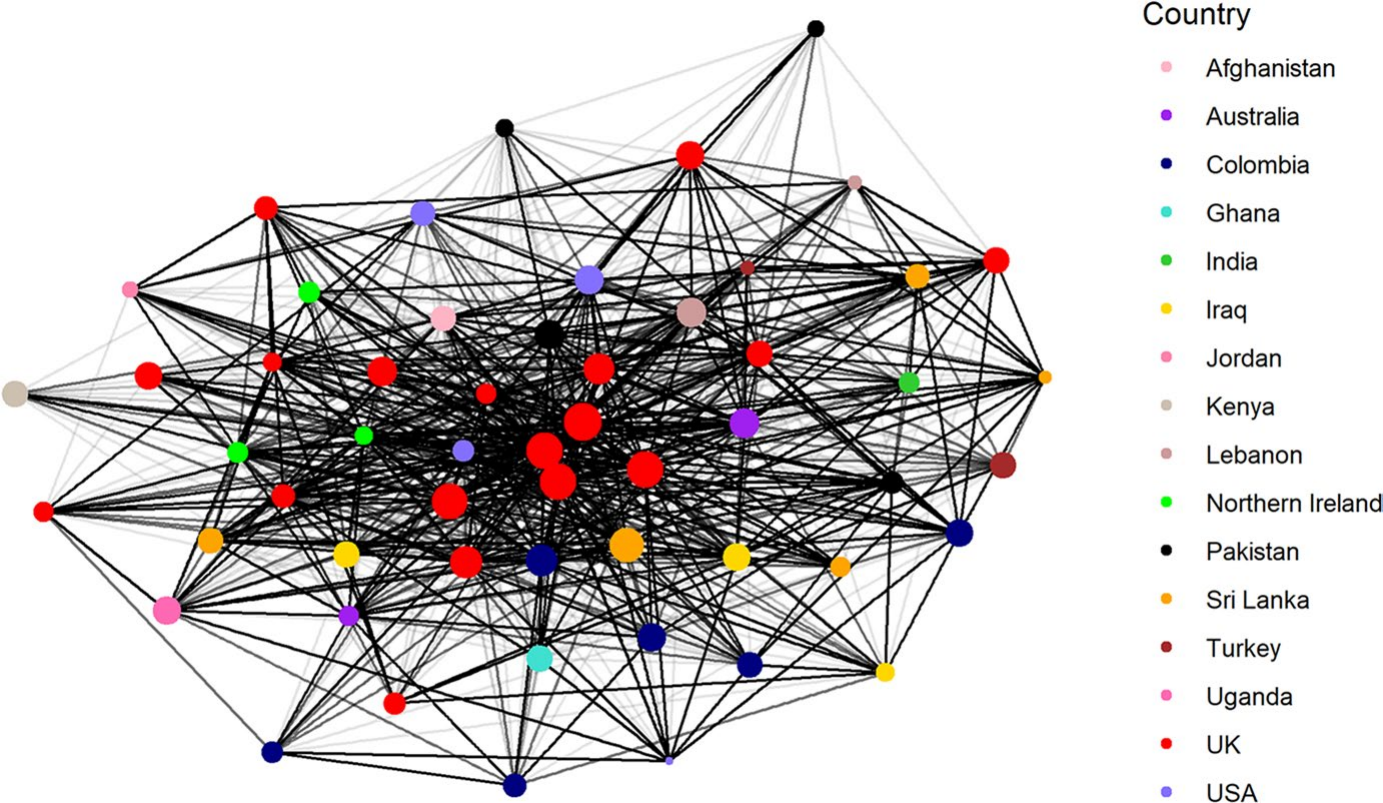
Network diagram showing all GJS Hub country networks

**Fig. 3:**
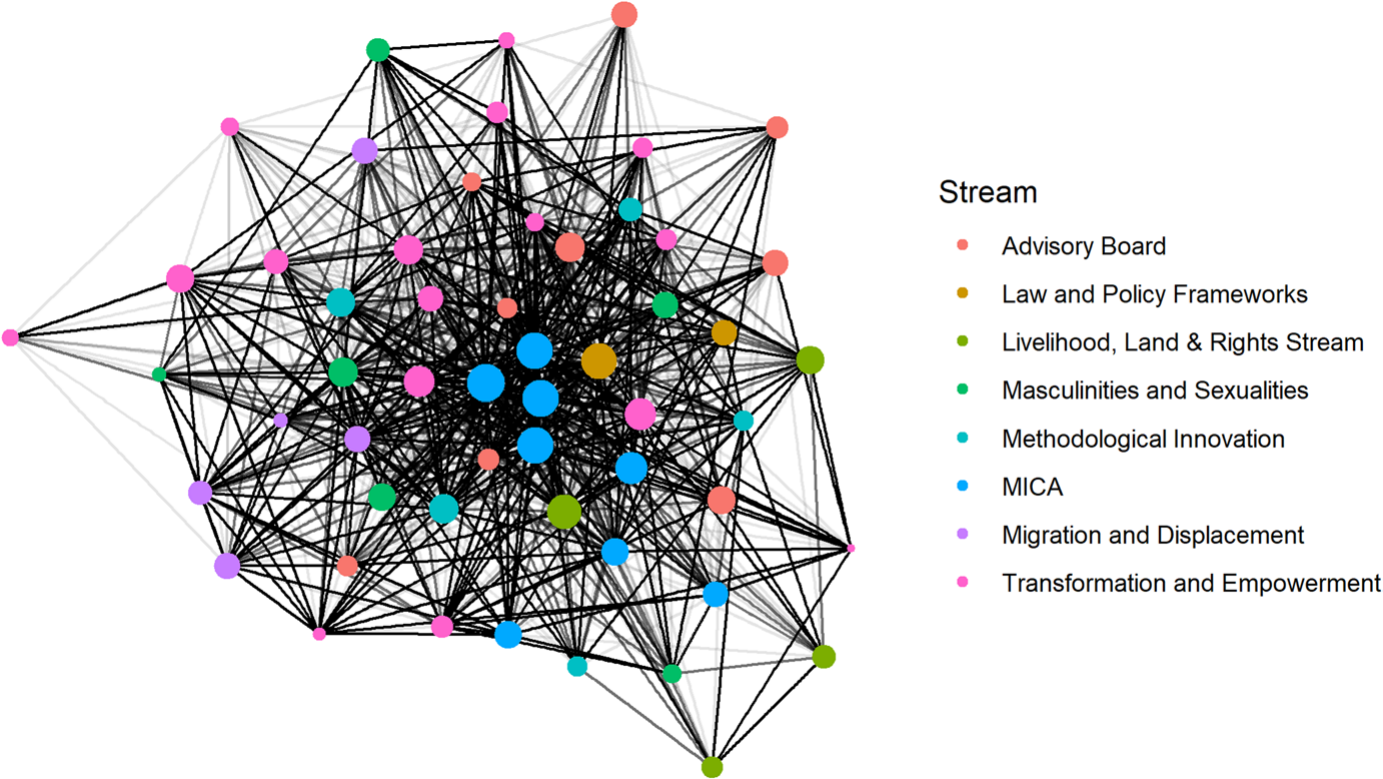
Network diagram showing all GJS Hub stream networks

The analysis shows that one year after the establishment of the Hub, it was a mildly centralised network whereby a small number of actors at the centre of the network have a large number of connections across the Hub, and a majority have a small number of connections across the network. We further looked at whether actors with similar levels of connectivity are more likely to connect with each other or if it varies depending on their centralisation (e.g. the core group has a lot of ties to less well connected actors but they do not connect well with each other). We found that the central group is very well connected across the entire Hub but the individuals outside the core group are not well connected to each other. This matches the expectation of how the setting up of the Hub would develop with the UK-based MICA team (see red dots in Fig. 2 and blue dots in Fig. 3) at the core. This small group of people (high degree actors), which also includes most members of the Executive Group (half of which are based in the UK), have connections to almost every individual in the network. We can see this confirmed in Fig. 2, which segments the network by country affiliation, clearly showing a core (red) group of UK-based Hub members. These findings confirm our initial design.

Regarding connections within the different thematic streams of the Hub, members who responded to the survey had more connections external to their stream than within (see Fig. 3). This is most likely the result of streams having been established by the core group of the Hub, rather than being pre-existing research networks who joined the Hub as a whole. At the time of the survey, streams had only met in person once, at the Hub Convention in January 2020.

Although we have only conducted one round of the survey so far, respondents could indicate if they had a connection with another member prior to the inception of the Hub and if so, how their relationship had changed over the last year of their joint Hub membership and what was driving this change. We found that 72% of tie changes were driven by the Hub (31% through the Convention and 41% through other Hub-related interactions). Roughly 60% of Hub members attended the in-person Convention in Sri Lanka in January 2020 and we would expect this to be the major event that introduced a lot of people to each other. In fact, 66% of respondents first met through a Hub-related activity. Apart from the Convention, members of the Hub mostly interact through their project work on the Hub, which most likely accounts for most of the ‘other Hub-related’ tie changes, but also, in the case of members of the management team and executive group, through regular meetings. Many members of the executive group were instrumental in putting together the initial application for the Hub and setting up its operational structures so we would expect their relationships to have strengthened during the first year of the project.

#### Reflections on Contribution of SNA for MEL

The interpretation of SNA results is based on several assumptions from network theory. The most important one being that it is a good thing if individuals in a network have many ties and are well connected to other members of the network. This assumes that everyone wants or *should* want to connect, network and collaborate. It does, in the first instance, not take into account whether there might be disciplinary, gendered, or geographical differences, which might, for example, encourage work in small, closely knit teams—maybe because extremely sensitive work is taking place in conflict contexts—rather than across the entire Hub. Some of the projects e.g. in Colombia, Iraqi Kurdistan, Sierra Leone, or Uganda rely on extensive networks across the Hub for comparative work and policy influence nationally and with international organisations—we would expect researchers and partners on these projects to be as connected as possible across the network. Other projects, e.g. in Afghanistan or Sri Lanka, due to the sensitivity of the work on gender in Afghanistan (even before the Taliban takeover in August 2021), or on human rights violations and post-conflict issues in Sri Lanka, might look to connect extensively with communities they are embedded and limit their interaction with foreign researchers and institutions as it might increase their exposure.

There are two stages of growing this network over the course of the Hub. First, the Hub itself as a network had to be established by facilitating connections and collaborations between different members of the Hub who are from different geographies, disciplines and professions. In the early years of the Hub, we would likely expect a fairly centralised network structure with the Principal Investigator (PI), Executive Group and the Management, Impact, Communication and Administration (MICA) team at its core. Those are the actors who were involved in the application stage and set up the governance structure of the Hub. The PI, MICA team and half of the Executive Group are Global North based and one aim of developing the Hub as a network is to decentralise it and facilitate more South-to-South connections. Hub members come from a variety of disciplinary perspectives, skill sets, career stages and geographic locations. The Hub model was designed to encompass feminist principles, which also includes an emphasis on collaborative working and ensuring that opportunities, including networking opportunities, are equitably distributed among all Hub members. Since the inception of the Hub and especially in preparation for and during the in-person Convention in January 2020, almost all communication happened over Microsoft Teams, a cloud-based team collaboration software. It was used as a collaborative platform for whole-Hub interaction, including to communicate information from the central team, but also for stream- and project-level interaction with some projects using it as their main platform for data storage and virtual meetings. It also allowed for the quick creation of new communication channels when members expressed an interest, e.g. in arts-based approaches. Since Teams was already established as a platform for communication and all Hub members had access to it prior to the COVID-19 pandemic, the move to online-only interaction was fairly smooth. During the pandemic, Conventions were moved online and included cross-project collaborations and presentations. In the later stages of the Hub and after the SNA was completed, we have moved to co-creating outputs with Hub members from different projects, streams and countries such as books, papers, documentaries, and trainings.

The SNA results allowed for the mapping of emerging relationships across the network and were designed to trace the development of relationships between and within groups including Global North and Global South partners, Early Career Researchers (ECRs) and more senior members, and practitioners and academics. Importantly, the SNA study aimed to provide tangible evidence on how networks like the GJS Hub can generate new insights through relationship development. The SNA would have been an important addition to surveys and anecdotal evidence in deciding where to target efforts of partnership building (as per objective iv). However, in the absence of this longitudinal evidence due to the interrupted funding mid-way, the ability to do this in an adaptive and tailored way was significantly hampered.

The second phase is to grow the Hub’s connections externally and link them to existing and emerging international networks and communities of practice on Women, Peace and Security, peacebuilding, International Law, and development. It would have been possible to identify which Hub members would benefit from a closer connection with external networks—that could then be fostered—and which networks to tap into at a Hub level to create the most impact, especially on a policy level. The connections built with these other networks are ultimately what will facilitate local and global policy change and institutional reform to advance gender justice and sustainable peace, building on new knowledge and advocacy networks, which amplify marginalised voices across different conflict contexts.

### Tomorrow’s Cities Hub

In a rapidly urbanising world, 60% of the area expected to be urban by 2030 remains to be built, opening a huge opportunity to build risk out of tomorrow’s cities. An initial assessment of the challenge showed that the disaster risk reduction community (including scientists, policymakers, development practitioners and business leaders) is currently operating in disconnected communities of practice and despite existing policy frameworks (e.g. Sendai Framework) approaches to disaster risk reduction are focussed on crisis management and not integrated into urban planning. The Tomorrow’s Cities Hub responds to this opportunity by working through research and development partnerships in four cities (Kathmandu, Nairobi, Istanbul and Quito). Its aim is to co-produce interdisciplinary research on multi-hazard risk working with stakeholders in order to influence disaster risk reduction policy and practice.

The co-produced research is implemented through a network consisting of 174 individuals from 54 partners including academic institutions, research centres, government departments and NGOs focussed in the four core cities. Given the aim of the Hub, strengthening capacity for interdisciplinary working as well as facilitating collaboration between researchers and stakeholders are critical to success. Monitoring how the hub network evolves through time, therefore, is a core component of the hubs MEL strategy. The drive for use of SNA as a tool for monitoring evolution of the hub network came from the evaluation and learning team, as part of a theory-based evaluation design. The intended users of the findings were the managers at central Hub level as well as in the city teams who were responsible for building an enabling environment for collaboration.

SNA was used as a baseline to understand the extent to which cross-collaboration was occurring across different attributes of individuals including their gender, career level and location in the initial phases of implementation. The expectation based on the Hub design was for an initial centralised network given the central UK-based leadership team had built the proposal through their own relationships with partners based in cities. Given the Hub’s commitment to equitable partnerships and to learn across the city contexts, we expected the network to evolve towards a less centralised structure through time. Intentionality in equitable partnerships and the potential of power asymmetries that funding structures across partners of the global North and South could reproduce the management and research structures were built to enable work across UK and cities. Collaboration was encouraged through formation of cross-hub thematic research groups, as well as building and supporting a network of ECRs to co-produce research outputs. Regular all hub meetings were convened online and plans for the first in-person all hub conference in 2021 was curtailed by the pandemic.

#### Methods

Data were collected between February and March 2021, through two databases. An administrative database with generic information about collaborators in the hub and an online survey through the SumApp platform generated a specialised survey to capture and visualise connections with other collaborators in the network. Our sampling framework included all individuals associated with the TC project at the time of the study. The total sample was 174 individuals and 53% made at least one connection (47% only show incoming ties and so we assume did not complete the survey) (for full details see Online Technical appendix). Self-identified female workers are slightly over-represented in the respondents (41% in respondents versus 23% non-respondents) which could account for more ties for women across the network, while ECRs are also slightly over-represented (47% respondents versus 37% non-respondents) which could also explain higher observed connections for ECRs overall.

#### Findings

**Fig. 4:**
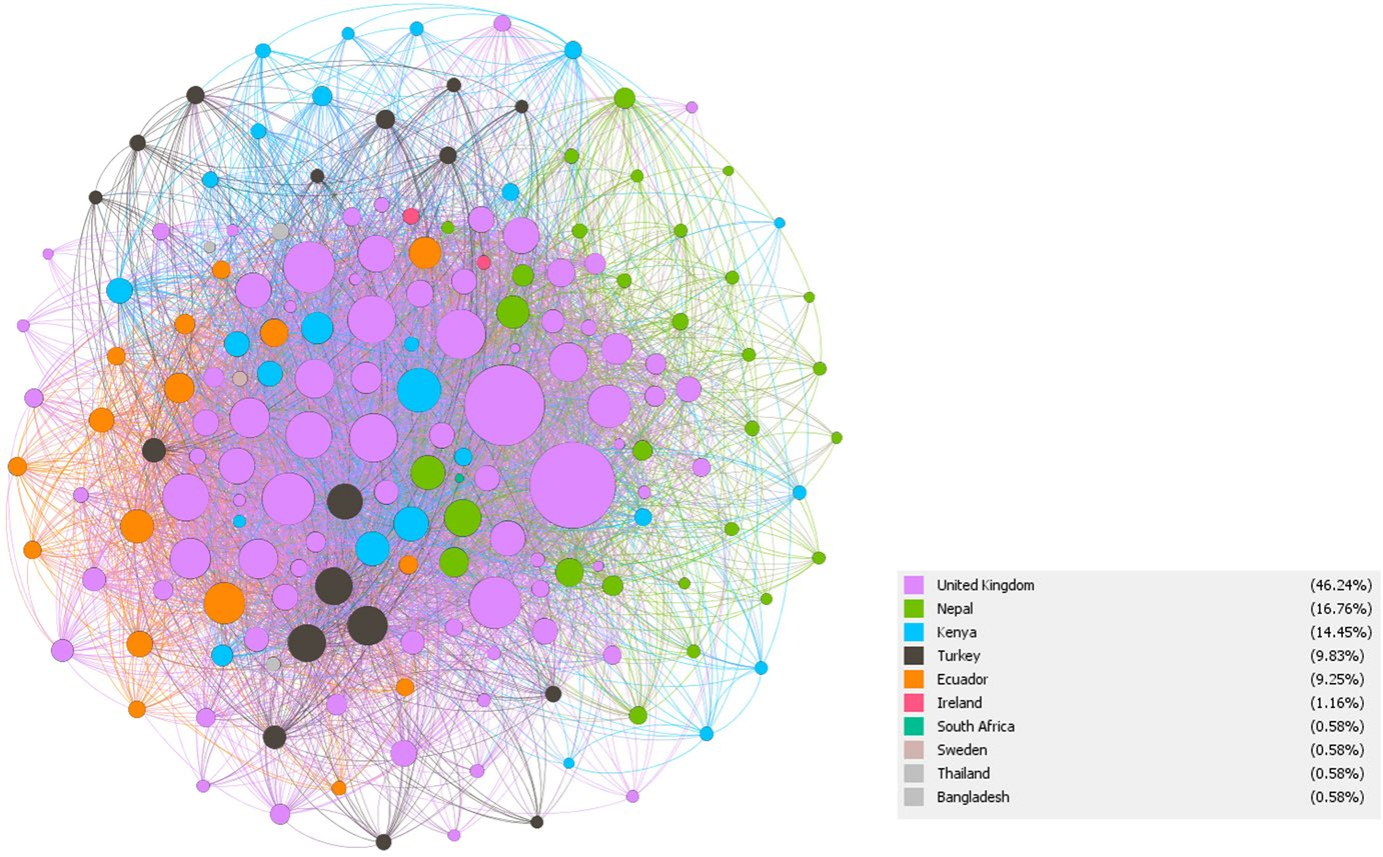
Network diagram showing all TC Hub geographic location networks

The analysis shows that after the establishment of the TC project, the network is fairly centralized. UK-based collaborators tend to be at the centre of the network and have a large number of connections across different locations—they are high degree actors. Yet, UK-based collaborators are also connected amongst themselves (purple nodes in Fig. 4). For example, connections realised (expressed as density) is twice as high among UK-based collaborators compared to those that work in LMIC countries. This shows that overall collaborations are initiated by the UK as the central hub whereby the four city networks tend to be connected through UK-based and cross-city affiliated individuals (shown in Fig. 4).

We then analysed collaborations between key attributes of location, career stage, gender and disciplines. We found that on average, individuals have 13 connections with those based at different locations. Yet only 9% (or an average of 1.2 connections) of those occur between LMIC-based collaborators (South–South collaboration). Collaborations are higher between UK-based members than between LMIC located members (an average of 9.5 versus 4.2 connections, respectively). It is more likely for those located in the same location to collaborate if they have a common contact. These findings were expected given the project design. The UK represents the biggest segment with 80 collaborators who are linked to one or more cities and organized around disciplinary/thematically focussed groups that co-designed research within and across disciplines. A greater extent of shared contacts within members in similar locations may further reflect historical collaborations and contextual knowledge held prior to establishing the new TC network.

**Fig. 5:**
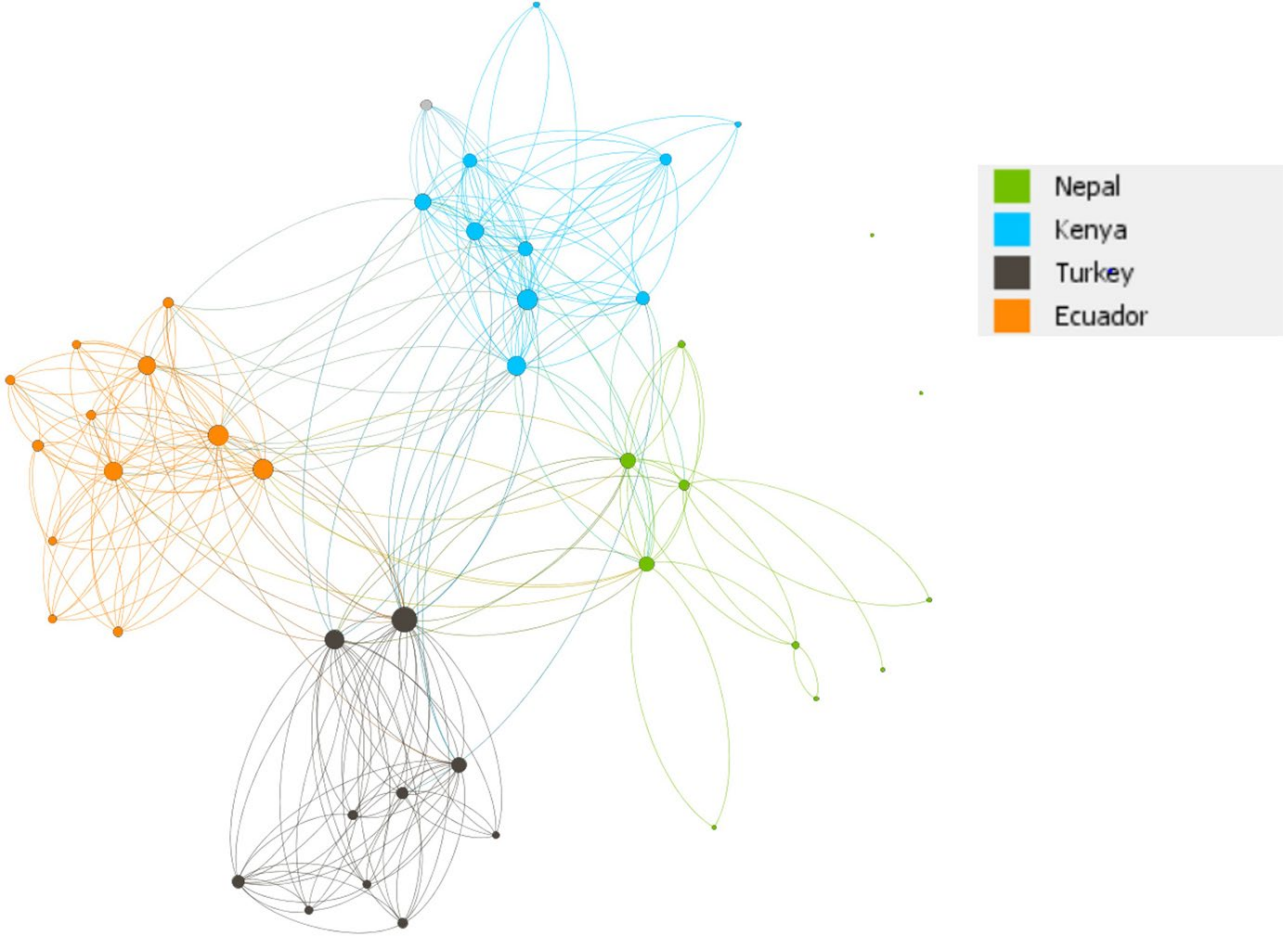
Network diagram showing collaboration among Early Career Researchers based outside of the UK for TC Hub

Collaboration also occurs among and across career stages. Peer-to-peer collaboration is more frequent overall among those at mid and senior career stages (an average of 17) that among those at early career stage (an average of 12). This could be explained in part by the fact that most of the 77 ECRs were independently recruited new hires in the TC project and so did not have existing connections to each other. Yet when looking at location, this pattern is different. For those based in the UK, peer-to-peer connection is higher among mid and senior career members (16 connections on average) than among ECRs (6 connections on average). And cross-career level collaborations are 8 connections on average. In LMIC locations, however, peer-to-peer collaboration among ECRs is more common (7 connections on average), while peer-to-peer collaboration among mid and senior researchers is lower (average of 4). Cross-level collaborations are 7 connections on average. What this shows is that peer-to-peer collaboration of ECRs is driven largely by their location as is illustrated in Fig. 5.

Lastly, regarding collaboration across genders, there are more connections among male collaborators overall (17 versus 12 among women). Yet, there is also collaboration between men and women (an average of 13 connections) overall. Again we found the pattern differed depending on location. Men located in the UK are more likely to collaborate with other men as compared to men located in LMIC. For instance, collaboration between men located in the UK is higher (on average by 6 connections) than between women located in the UK. In LMIC, this is overall more balanced. This suggests that gender influences how much members collaborate within their peer group.

#### Reflections on Contribution of SNA for MEL

This application of SNA was part of a staged and modular theory-based evaluation design that aimed to explore a core assumption of the hub’s theory of change around interdisciplinary working and equitable partnerships. The baseline application described the network at the outset in order: (i) to identify opportunities to enhance interdisciplinary and equitable partnerships as part of adaptive programme management; and, (ii) to inform impact evaluation design to assess the contribution of network collaborations to achieving intended shifts in urban planning (evaluating relationships beyond the hub).

Visualization of the network overall and specific visual patterns in collaboration did enhance understanding of how the initial project design is reflected in the social fabric of collaboration. The fairly centralised initial network built confidence in the initial design with a large UK-based central team, and an explicit intent to build city-focussed research partnerships. While we did not have an ‘ideal network’ structure against which we intended to monitor the evolution of the network, we did anticipate that through time we would see greater collaboration between the non-UK-based members, in line with the intention to build equity in the partnership.

The analysis also enabled observation of unanticipated dynamics—such as gendered dynamics of collaboration between UK and non-UK-based individuals, and the marked difference between the way ECRs and more senior individuals are connected across the network. These structural patterns revealed would need to be deepened through focus groups with hub collaborators to explore the drivers behind them and identify potential programme adaptations in line with the goal of moving towards a network structure with greater connections across more members. Visualising these patterns could also support existing conversations within the Hub around undertaking gender bias and power training as signalled by at least one of the city leadership teams as a priority. In this way, the application of SNA has shown potential to produce learning that could influence the next phases of work to support collaboration in the Hub.

The application of these findings for decision-making processes, however, depends in large part on the quality of data and resulting findings. Data limitations are a common challenge in SNA (e.g. Wasserman and Faust 1994; Newman 2003) due to its exponential growth of observations and complexity. Further, SumApp required respondents to scroll through a list of all 174 people in the Hub which could result in biases, e.g. individuals mentioned towards the end could be less frequently selected due to response fatigue. This and other sources of response biases (which are common in SNA) influence the extent to which these findings constituted actionable learning for the Hub.

## Findings from Cross-Case Analysis

As R4D programmes, all three Hubs set out to intentionally build collaboration across disciplines, geographies and hierarchies. All cases share the dual (and interconnected) objectives of (i) using SNA to monitor progress of their intended designs through describing and tracking how collaborative relationships change over time across significant attributes, and (ii) using the visualisations and resulting appreciation of the structure of the network to influence its development in intentional ways—to support ‘network weaving’ (Vance-Borland and Holley 2011). Our experiences are from the early phases of implementation. While we do not discuss resulting adaptations, we do reflect on the opportunities for responding to learning that emerged when using SNA as a learning and an evaluation tool.

### Data Challenges and Respondent Bias

The practical challenges of data collection and analysis in SNA are well described in the literature and relate, among other factors, to the extensive time required to respond to lengthy surveys leading to incomplete data sets with consequent implications for rigorous understandings of whole networks (e.g. (Newman 2003; Penuel et al. 2006; Popelier 2018). Particularly relevant to applications within evaluation are threats to construct validity that result from ambiguity in how relational attributes are collected (how the type and strength of collaboration is described) and relatedly, how these are interpreted (Popelier 2018). Incomplete datasets can lead to weak ties being under reported, influencing the overall validity of findings.

Consequently, perhaps the most important step in the design of a SNA study in the context of evaluation is defining the ties, or connections, at the outset. This requires clarity of the aspects of collaboration that are of interest to the study, as well as knowledge of how these will be interpreted by respondents. All our cases are large networks (with over 100 members), and given the novelty of using SNA to explore relational aspects of research projects, choosing where to focus had to align with key evaluation and learning demands. As shown in Table 1, the relational attributes used in each case were driven by the specific and different evaluation objectives of each—OHPH mapped Hub-related collaborations (including research, outreach, administrative activities), GJSH mapped both the strength (light, good, strong, none) and origin of connections (non-Hub, Hub, specific Hub activity), while TCH mapped both formal and informal interactions within the hub through strengths (in four categories).

OHPH members were asked with whom they had worked on a range of activities over pre-defined periods of time. However, the interpretation of the level of engagement which qualifies as “working together”, as well as the definition of a specific task is likely to vary among respondents. In the TCH case, four categories or strengths of collaboration were used, but interpretations of each cannot be assumed to have been uniform. Given the size of the partnerships and the period of time covered by each survey, especially in the case of OHPH, recall bias cannot be excluded. Attempts were made to minimise them by including the list of all members in the questionnaire (and TCH and GJS included photos), so respondents were less likely to forget collaborators.

In spite of these efforts, unsurprisingly, missing data was a challenge in all three cases. This was dealt with in different ways. OHPH avoided under estimating connections by examining only partial networks, TCH used the reconstruction method (Liu et al. 2019; Stork and Richards 1992) and assumed incoming ties are reciprocal for non-respondents in order to examine the full network, and GJSH examined both the full network (including non-respondents) and the partial network using the listwise approach (Pepinsky 2018). Complex model-based approaches, such as Baysean models, are gaining popularity, but are often difficult to implement (requiring a complex model to be specified and estimated), and can result in introducing other forms of bias by imputing edges that over generalise the tendencies observed in other parts (i.e. information rich areas) of the network (Smith et al. 2022). In the context of SNA for learning, these more complex modelling strategies were not considered worth the additional time and effort. As we discuss later, there are inherent limits to what SNA can reveal on its own, and as part of broader MEL strategies, triangulation with other methods we posit is a better approach to mitigating the challenge of missing data.

An obvious yet not insignificant response to overcoming the challenge of incomplete network data is to invest early in strategies to increase the proportion of Hub members participating in the survey. The TCH chose to use the SumApp survey in order to turn the SNA process into an explicit network weaving exercise, with the assumption that this would motivate hub members to respond. SumApp creates a personal profile for each hub member with a unique URL and users can visualise the network real time as respondents update their connections (while the application is ‘live’). Feedback from ECR members suggests that this was indeed motivating for many of them because it aligned with their motivation to network within a large hub. Yet this did not necessarily hold true for other members of the Hub. Understanding what might motivate greater response, therefore, is an important step in planning SNA as a learning and network weaving tool.

## Challenges of Interpretation

The challenge of confirmation bias in interpreting SNA findings in the context of programme evaluation is well described in the literature (e.g. (Popelier 2018)). Critics argue that limited ability to objectively interpret the results may lead to an interpretation which aligns with the investigators’ (and/or programme managers’) preconceived ideas rather than taking the data at face value, or indeed, pretending that an ‘objective’ interpretation exists. This follows a gold standard view in evaluation that confirmation bias is to be avoided at all costs. In contrast, employing complexity-aware evaluation designs, we were working within programmes implementing SNA as a participatory and learning-oriented evaluation method, working with (rather than controlling for) the experiences and aspirations of those involved in programme design (Apgar and Allen 2021). Interpretation of the findings, therefore, required the situated experiences of programme implementers and we embraced their interpretations (which are inherently biased) as an important explanatory device.

As (Durland and Fredericks 2005) note the importance of specific network information should be seen as relative to programme needs at that particular time, embracing internal interpretation as the principle goal. In all three cases, the baseline application of SNA served as a useful empirical check on how the initial programme design was reflected in the social fabric of collaboration. The network structures that became visible at the initial phase were interpreted based on our expectations given the intentional designs. Table 2 provides a comparative view across the three cases. In all three cases, the network structures revealed in the early stages of the Hubs matched the expectation of centralised structures, based on their set up driven by the parameters of the funding set by UKRI. In all cases UK-based research leaders built the Hub networks initially through contracting partners and researchers based in countries of focus or operation.

**Table 2:** Comparison of SNA findings

Hub	Observed network structure versus expected network structure	Explanations
One Health Poultry Hub	An increase in connectedness across the first two time periods was expected, while a decrease in connectedness between the second and third time periods was not expected. Collaboration was found to be more likely between collaborators in the same country.	Disruption to collaboration through the pandemic led to the inability of network members to engage in facilitated face-to-face interactions While interactions across the consortium were thus more difficult, the implementation of project activities within each study country meant that collaborations were more active within rather than between study countries
Gender, Justice and Security Hub	After one year of implementation, as expected, centralised network members were well connected to all actors with some having connections to all Hub members. As envisioned, new collaborations were established with members outside of each thematic group (or stream) due to Hub activities. Greater collaborations with Hub members within and outside of each thematic group (or stream) would have been expected.	Streams were set up as new collaborations by UK-based members rather than being formed around pre-existing collaborations
Tomorrow’s Cities Hub	After one year of implementation the Hub was centralised, with UK-based collaborators at the centre, confirming the initial design. Collaboration between ECRs was found to be driven largely by where they are located rather than disciplinary or thematic research groupings. Further, gender influenced how much collaboration happens within peer groups and teams in different locations.	The city-focussed research design of the Hub necessarily required within location collaboration for integrated implementation and impact planning Based on understanding built through reflection on gender and power awareness in the Hub, the gendered dynamics of collaboration can be explained by the different approaches to gender taken by leaders across research themes and locations

As others have shown, it is the repeated applications of SNA that enables a picture of evolution and change through time and brings it to life as a monitoring tool (Aboelela et al. n.d.; Provan et al. 2005). Yet as social networks are living and constantly evolving systems, we expect that they will organically shift in time and some of their dynamics will be unpredictable. This leads us to ask—*how should we interpret the changes as part of monitoring the network?* Some argue that lack of an ideal network structure means there cannot be standard benchmarks for judging performance through use of SNA and this weakens its evaluation potential (e.g. (Haines et al. 2011)).

Our intention was not to monitor progress against an ideal structure, but rather, to iteratively learn with and as the structure evolves. But assumptions from network theory are, often implicitly, applied. For example, assuming that a network is ‘stronger’ when individuals have many ties and are well connected to other members of the network. In large networks, such as R4D collaborations, however, it may in fact be that specialisation, or particular geographic clustering within the network is optimal for achievement of desired goals. Interpretation of the structure, then, must follow the intention of the network, which in turn is always situated in a particular moment and context. Our cases illustrate such a situated contextual approach to using SNA.

In all cases, we held assumptions about a desirable evolution of the hub structure in line with the stated goals of equity across specific hierarchies of power (such as gender, Global North-Global South, career level) (see Table 2 for observed versus expected network structures). These assumptions are aligned with the theory of change of the GCRF funding mechanism overall and the programmes specifically. The OHPH interpreted their findings based on an assumed evolution along a spectrum—a hypothetical star network (with the PI being connected to everyone and no link between others) at one end, and a complete network (with everyone connected to each other) at the other end. Indeed the SNA across time periods revealed an unexpected surge in centralisation in the last study period which suggested movement in the opposite direction. These observed trends in network development could be related to the move from face-to-face to online interactions due to COVID-19, adding some confidence that they are reflective of ‘actual’ processes. This finding can help to identify individuals between which collaborations should be fostered, and support thinking further about how the hub could recover post-pandemic to continue to build collaboration in the ways it intended.

The GJSH captured connections prior to the inception of the Hub (retrospectively) and then 16 months into the project (time of the survey). This showed how the Hub was set up and how members started to connect. The importance of in-person all-Hub Conventions was underlined as it was both a key event where people first met each other as well as a key driver of strengthening connections. Over the course of the pandemic, similar to the OHPH, Conventions moved online and while this continued the opportunity to meet officially, it greatly reduced the chances for spontaneous interactions which are most likely to strengthen relationships.

What our experiences suggest is that whether implicit or explicit, the programme designs were manifest through the baseline and subsequent application in a way that made them visible. This offered the opportunity to challenge earlier assumptions and to identify hidden or unexpected dimensions that warrant further exploration, as well as mechanisms to nudge the network in desirable directions. In the case of the TCH baseline, we saw that even in the early stages some unexpected dynamics were revealed—such as collaborations between female members based outside of the UK being greater than for female members based in the UK. The TCH had procedures for reflecting on the ways in which power imbalances influenced internal team dynamics and how equitable decision making was. This was part of a broader intention to work in equitable ways (Snijder et al. this issue). Revealing the gendered dynamics of collaboration could add further weight to the requests of some city management teams to provide gender sensitivity training to all network members. It also allowed questioning an underpinning (positivist) premise of ‘more is better’. Starting with a deliberate project design as we did, network studies as an M&E tool offer the opportunity to re-evaluate and -classify measures from an aspect of applicability for the ‘ideal function’ rather than ‘ideal structure’ of a network.

### Strengthening Causal Inference

The ways in which SNA can support causal inference are still debated within the evaluation literature, and many applications of SNA still struggle to determine causal relationships between internal network structure and external network outcomes. In the context of evaluating R4D programmes, being able to make this link is important to add weight to SNA as an evaluation method. The structural paradigm of SNA suggests that structural mechanisms influence how changes unfold, yet the relational aspects are so many, indeed potentially infinite, that establishing clear causal links is challenging (Doreian 2001; VanderWeele and An 2013). A response to this dilemma is to situate the structural analysis within a contextualized theory about how the causal inference is hypothesized (a causal theory of change).

As discussed above, all three of our SNA cases were part of broader evaluation designs. While all three are focussed on visualising and describing the ‘internal’ networks, in TCH and GJSH, the intention (prior to funding cuts) was to use understanding of how the internal network is working (and evolving through time), alongside other evaluation research on equitable partnerships to build contribution claims around how the network structure (and ways collaborations are taking shape across hierarchies and power structures) contributes to intended programme outcomes. In the case of TCH, the focus of external interactions is on influencing the co-production of risk informed urban planning, while in the case of GJSH, the focus is on the end goal of shifting patriarchal modes of knowledge production on sustainable peace. In this way, using SNA as a monitoring tool can not only build a picture of how the internal structure is evolving but also can offer data points to support theory-based evaluation.

Additional methods are required to enhance interpretation and reveal the meaning given to relations identified and so to crystalise key causal mechanisms for evaluation to investigate further. Often quantitative SNA is complemented with qualitative approaches (Hopkins 2017; Kolleck 2016). All three Hubs have multiple other sources of monitoring data that could be used to supplement the SNA. TCH, for example, developed outcome case studies for monitoring outcomes, and implemented a survey on interdisciplinary working to shed light on some of the patterns revealed. For example, an outcome case study on interdisciplinary working in Quito evidences how intentional reflection on ways of working and building of interdisciplinary capacities within the team has opened up opportunities for greater engagement with local partners for multi-hazard risk research. This qualitative analysis can support causal claims around how collaboration between team members from different disciplines (visualised through SNA) which produce internal outcomes (such as openness to participatory methods) relates to movement along a desired pathway towards equitable outcomes. While SNA is not a causal methodology, it can provide evidence of collaboration and thus can confirm or disconfirm achievement of early and internal desired outcomes in the way the programme (network) is set up to deliver impact.

### Ethical Dilemmas in SNA for Evaluation

Making visible how individuals are interacting with colleagues or partners comes with ethical challenges and risks if the results are interpreted as judgements of individual performance (Kadushin 2005; Penuel et al. 2006). The GCRF Hubs were funded to intentionally support collaboration across established hierarchies of power, turning collaborative behaviours into expectations. Anonymization, which is the standard approach to managing research ethics and minimizing risk to participants, is often not possible and usually not desirable when using SNA to intentionally support network weaving.

The GJS Hub experienced a concern that members might alter how they reported a connection based on a perception of how socially acceptable that connection might be. Of course any reported connection is a subjective measure of that individual’s perception of a relationship but there is still likely to be systematic under- or overreporting. For example, it is likely that an ECR might underreport connections with more senior scholars so as not to assume a ‘strong’ relationship when that perception might not be reciprocal. Similarly, there might be cultural differences in perceptions of relationship strength, where, for example, a US-based member of the Hub might consistently rate relationships as stronger than UK-based Hub members.

As discussed above, the TCH used the SumApp online tool to support intentional network weaving which was experienced as motivating for ECRs. On the other hand, accessibility of all connections to everyone in the Hub might have deterred some from reporting for similar reasons described for GJSH. Visualising your own position in a network that aspires to be collaborative could, therefore, be experienced as positive or negative depending on how connected you are and how much you value connection. One advantage is that individual learning, and first person reflection, is made possible through seeing oneself as part of the network. But this requires network members to value this capability for self-reflections above any concerns they have about being judged based on their position in a network.

## Lessons for Future Use of SNA in Evaluation of R4D Programmes

Across the three cases of SNA in evaluation of large R4D programmes, we have illustrated that in spite of the challenges with data and interpretation (which are common to SNA), it is useful as a monitoring tool when used to reflect on underlying assumptions about collaboration and resulting network structures. From our analysis we conclude with three lessons for future use of SNA within evaluation of R4D programmes.

1. The more explicit assumptions about collaboration are at the outset, the more useful the empirical view of collaboration revealed is to programme learning. A contextualized theory of collaboration could be created at the outset to guide the SNA study. This is in line with Davies’ (2009) call for a theory-based and deductive approach to SNA in evaluation.
2. Combining SNA with other methods can enhance interpretation and reveal the meaning given to structural views. This can strengthen causal inference about relational causal mechanisms making SNA a necessary, but not sufficient method to evaluate R4D programmes.
3. Navigating the challenges of interpretation and ethical dilemmas requires careful consideration as well as an enabling institutional and political environment for use of SNA to support learning. Embedding the interpretation of SNA findings within participatory learning moments (such as after-action reviews) would strengthen the use of SNA findings in learning-oriented evaluation design, as suggested by others (Drew et al. 2011; Durland and Fredericks 2005)

## Supplementary Information

Below is the link to the electronic supplementary material.

Supplementary file1 (DOCX 244 KB)

## Footnotes

1 https://www.ukri.org/our-work/collaborating-internationally/global-challenges-research-fund/.

2 The six research streams are thematic areas of work (Law and Policy Frameworks; Livelihood Land and Rights; Masculinities and Sexualities; Methodological Innovation; Migration and Displacement; Transformation and Empowerment).
