# Supplementary material for: Revealing the Relational Mechanisms of Research for Development Through Social Network Analysis
Source: Eur J Dev Res. 2023 Jan 25;35(2):323–50. doi: 10.1057/s41287-023-00576-y (PMC9875764; doi:10.1057/s41287-023-00576-y)
Supplement: Supplementary file 1 — Supplementary file1 (DOCX 244 KB) [file 41287_2023_576_MOESM1_ESM.docx]

# Technical Appendix

## **One Health Poultry Hub Full Technical Case Study**

### **Study design and data**

Surveys were built using the Jisc Online Surveys tool. Invitations were mailed out to 138 co-investigators and researchers engaged with the OHPH, contracted research staff, postgraduate students and managerial staff for the first survey (March 2020), and 182 people for the second survey (February 2021). Overall, 202 individuals were invited to participate. Reminders were sent to non-respondents approximately a fortnight after opening the surveys, and the surveys were closed after about a month. 66 people responded to the first survey (48%) and 81 people responded to the second survey (45%). 40 people responded to both surveys. Data were extracted in CSV format and all SNA was carried out in R statistical software.

### **Methods**

Unweighted and undirected networks were built for each of the three periods (P0, P1 and P2), with participants as nodes and active collaborations as edges. Respondents who did not report any connection to any other OHPH people over the periods of interest were excluded. Two sets of nodes were computed for each period: all respondents (period-specific networks), and respondents who completed all three questionnaires (cohort networks). Network metrics were computed to assess the network connectedness, and whether node-level factors (i.e. country, discipline, career stage and gender) were associated with the occurrence of edges between nodes.

### **Results**

The size of the period-specific networks ranged from 58 to 81 nodes. The cohort networks had 37 nodes. About two thirds of respondents were from the study countries, and almost all others were based in the UK. Most respondents were male, biological scientists, and at mid to late career stage (Table 1).

Table 1: Characteristics of respondents including in the period-specific and cohort networks. Periods P0, P1, P2: before the Hub, first and second year of the Hub; cohort: respondents completed all three questionnaires; ^1^Australia, Belgium, France and Hong Kong SAR; ^2^Administration, communication, pedagogy.

|  | **P0** | **P1** | **P2** | **cohort** |
| --- | --- | --- | --- | --- |
| **Number of nodes** | **58** | **59** | **81** | **37** |
| *Country* |  |  |  |  |
| Study countries | 40 (69%) | 41 (69%) | 49 (60%) | 26 (70%) |
| Bangladesh | 11 (19%) | 11 (19%) | 15 (19%) | 11 (30%) |
| India | 17 (29%) | 18 (31%) | 18 (22%) | 9 (24%) |
| Sri Lanka | 4 (7%) | 4 (7%) | 6 (7%) | 2 (5%) |
| Viet Nam | 8 (14%) | 8 (14%) | 10 (12%) | 4 (11%) |
| Non-study countries | 18 (31%) | 18 (31%) | 32 (40%) | 11 (30%) |
| United Kingdom | 17 (29%) | 17 (29%) | 28 (35%) | 11 (30%) |
| Others^1^ | 1 (2%) | 1 (2%) | 4 (5%) | 0 (0%) |
| *Gender* |  |  |  |  |
| Female | 23 (40%) | 24 (41%) | 37 (46%) | 12 (32%) |
| Male | 35 (60%) | 35 (59%) | 44 (54%) | 25 (68%) |
| *Discipline* |  |  |  |  |
| Biological sciences | 38 (66%) | 38 (64%) | 54 (67%) | 24 (65%) |
| Social sciences | 10 (17%) | 10 (17%) | 13 (16%) | 7 (19%) |
| No research^2^ | 10 (17%) | 11 (19%) | 14 (17%) | 6 (16%) |
| *Career stage* |  |  |  |  |
| Early career | 16 (28%) | 17 (29%) | 38 (47%) | 12 (32%) |
| Mid/late stage | 42 (72%) | 42 (71%) | 43 (53%) | 25 (68%) |

Table 2 shows that each period-specific and cohort network was a connected component and showed a high small-world index^[[1]](#footnote-1)^ with a clustering coefficient much higher than, and an average path length close to, those estimated for random networks with the same number of edge and nodes^[[2]](#footnote-2)^. From P0 to P1, the density and clustering coefficient increased (degree centralisation index), and the diameter and average path length decreased (betweenness centralisation index), signifying that this increase in connectedness was distributed among Hub partners. However, this trend was reversed from P1 to P2. This pattern was observed in period-specific and cohort networks (Table 2).

Table 2: Characteristics of the networks. The median value of the clustering coefficient and average path length in the simulated random networks and their associated p-value are shown in brackets.

|  | Period-specific networks | | | Cohort networks | | |
| --- | --- | --- | --- | --- | --- | --- |
|  | P0 | P1 | P2 | P0 | P1 | P2 |
| Density | 0.16 | 0.29 | 0.23 | 0.22 | 0.35 | 0.32 |
| Diameter | 4 | 3 | 4 | 4 | 3 | 4 |
| Clustering coefficient^1^ | 0.44 (0.16, p<0.001) | 0.57 (0.29, p<0.001) | 0.49 (0.22, p<0.001) | 0.55 (0.22, p<0.001) | 0.61 (0.34, p<0.001) | 0.55 (0.32, p<0.001) |
| Average path length^1^ | 2.2 (2.0, p=1) | 1.8 (1.7, p=1) | 1.9 (1.8, p=1) | 2.1 (1.9, p=1) | 1.8 (1.7, p=1) | 1.8 (1.7, p=1) |
| Small-world index | 2.8 | 1.9 | 2.1 | 2.4 | 1.7 | 1.7 |
| Degree centralisation | 0.47 | 0.41 | 0.5 | 0.39 | 0.43 | 0.48 |
| Betweenness centralisation | 0.27 | 0.06 | 0.12 | 0.27 | 0.09 | 0.15 |

To assess the possible influence of node-level factors (country, discipline, career stage, gender) on edge distribution, we computed the density of edges within and between groups of nodes defined by these factors, and compared them to the null density distributions estimated through permutations (i.e. under the null hypothesis: no association between edges and factor of interest)^[[3]](#footnote-3)^ (Table 3). For all periods and network types, the likelihood of a connection increased if two partners were from the same country, but decreased if they were both from different study countries. By P2, all UK and 82% of study country partners were engaged in connections between the UK and study countries, whereas connections across study countries involved 47% of study country partners. Density of edges was higher among social scientists, mid/late career, male partners, and lower among female partners embedded in the P2 period-specific network than expected by chance. These associations were not, however, found in the cohort-specific networks.

Table 3: Density within and between groups. ^1^The median value of the density in their permuted networks and the associated p-value are shown in brackets.

|  | Period-specific networks | | | Cohort networks | | |
| --- | --- | --- | --- | --- | --- | --- |
|  | P0 | P1 | P2 | P0 | P1 | P2 |
| *Country* | | | | | | |
| Within: UK | 0.35 (0.15, p=0.002) | 0.60 (0.29, p<0.001) | 0.41 (0.23, p=0.001) | 0.36 (0.22, p=0.06) | 0.58 (0.35, p=0.02) | 0.53 (0.31, p=0.048) |
| Within: each study country | 0.49 (0.16, p<0.001) | 0.56 (0.29, p<0.001) | 0.56 (0.22, p<0.001) | 0.68 (0.21, p<0.001) | 0.70 (0.35, p<0.001) | 0.74 (0.33, p<0.001) |
| Between: study countries | 0.03 (0.16, p<0.001) | 0.10 (0.29, p<0.001) | 0.03 (0.22, p<0.001) | 0.07 (0.22, p<0.001) | 0.15 (0.34, p<0.001) | 0.08 (0.33, p<0.001) |
| Between: UK-study countries | 0.11 (0.16, p=0.002) | 0.31 (0.29, p=0.31) | 0.23 (0.23, p=0.46) | 0.15 (0.22, p=0.02) | 0.34 (0.35, p=0.39) | 0.34 (0.33, p=0.35) |
| *Discipline* | | | | | | |
| Within: Biological | 0.19 (0.17, p=0.28) | 0.28 (0.31, p=0.16) | 0.23 (0.24, p=0.29) | 0.19 (0.21, p=0.26) | 0.31 (0.33, p=0.33) | 0.31 (0.32, p=0.45) |
| Within: Social | 0.36 (0.16, p=0.04) | 0.69 (0.29, p=0.003) | 0.67 (0.24, p<0.001) | 0.24 (0.19, p=0.42) | 0.33 (0.33, p=0.50) | 0.33 (0.33, p=0.51) |
| Between: Biological-Social | 0.12 (0.17, p=0.04) | 0.32 (0.31, p=0.44) | 0.22 (0.24, p=0.28) | 0.24 (0.21, p=0.23) | 0.36 (0.33, p=0.29) | 0.32 (0.32, p=0.46) |
| *Career stage* | | | | | | |
| Within: Early | 0.12 (0.15, p=0.28) | 0.17 (0.29, p=0.049) | 0.19 (0.22, p=0.19) | 0.20 (0.21, p=0.46) | 0.49 (0.33, p=0.10) | 0.56 (0.32, p=0.02) |
| Within: Mid/late | 0.21 (0.16, p=0.004) | 0.37 (0.29, p=0.006) | 0.30 (0.22, p=0.02) | 0.23 (0.22, p=0.38) | 0.33 (0.34, p=0.39) | 0.26 (0.32, p=0.10) |
| Between: Mid/late-Early | 0.10 (0.16, p=0.001) | 0.22 (0.29, p=0.005) | 0.20 (0.23, p=0.001) | 0.21 (0.22, p=0.35) | 0.33 (0.35, p=0.30) | 0.33 (0.33, p=0.41) |
| *Gender* | | | | | | |
| Within: Female | 0.16 (0.15, p=0.48) | 0.26 (0.29, p=0.30) | 0.16 (0.23, p=0.04) | 0.18 (0.21, p=0.38) | 0.29 (0.33, p=0.33) | 0.35 (0.32, p=0.43) |
| Within: Male | 0.18 (0.16, p=0.25) | 0.35 (0.29, p=0.08) | 0.30 (0.22, p=0.01) | 0.19 (0.22, p=0.25) | 0.36 (0.34, p=0.38) | 0.28 (0.33, p=0.18) |
| Between: Male-Female | 0.14 (0.16, p=0.10) | 0.26 (0.29, p=0.02) | 0.21 (0.23, p=0.01) | 0.25 (0.22, p=0.07) | 0.34 (0.35, p=0.42) | 0.36 (0.33, p=0.10) |

We then assessed whether the centrality of a node was associated with its characteristics (country, discipline, career stage, gender) using multivariable permutation-based linear models^[[4]](#footnote-4)^. We considered two centrality measures: degree, the number of other nodes with which a node was connected, and the betweenness, the extent to which a node lay on the shortest path between two others. For the period-specific network at P2, there was weak evidence that the average degree could be lower for study country than for UK partners (-27%, p=0.06), and for women than for men (-24%, p=0.08). Such an association was not observed on the cohort network. Betweenness was not associated with any abovementioned node-level factors.

**Gender Justice and Security Hub Full Technical Case Study**

**Study design and data**

In order to capture the complete network of the Gender, Justice and Security Hub, we utilised a nominalist strategy to set the boundary of the network, where we surveyed all members of the UKRI GCRF Gender, Justice and Security Hub (Laumann, Marden, & Prensky, 1983, Knocke & Yan, 208).  Exploring a complete network allowed us to visualise the overall structure of the network, including density and the strength of connections between members. In addition, we sought to explore types and patterns of connections within the network, by looking at connections by attributes such as geography, career-stage and by Hub project.

Data was collected between June - December 2020. Our sampling framework included all individuals associated with the Gender, Justice and Security Hub at the time of the study. The total sample was 121 individuals, the partial network consists of 55 individuals. Respondents were primarily from the UK, female and between 25-54 years old and academics (shown in Table 4).

Table 4: Table of number of individuals belonging to each Stream and Country with proportion of respondents from each Stream and Country.

| Group | | Total Count | Percentage of group that responded | Percentage of respondents from group |
| --- | --- | --- | --- | --- |
| Stream | Advisory Board | 17 | 17.65 | 5.45 |
|  | Law and Policy Frameworks | 6 | 66.67 | 7.27 |
|  | Livelihood, Land & Rights Stream | 9 | 88.89 | 14.55 |
|  | Masculinities and Sexualities | 12 | 41.67 | 9.09 |
|  | Methodological Innovation | 8 | 75 | 10.91 |
|  | MICA | 9 | 88.89 | 14.55 |
|  | Migration and Displacement | 22 | 45.45 | 18.18 |
|  | Transformation and Empowerment | 38 | 28.95 | 20.00 |
| Country | Afghanistan | 1 | 0 | 0 |
|  | Australia | 2 | 50 | 1.82 |
|  | Canada | 1 | 100 | 1.82 |
|  | Colombia | 8 | 75 | 10.91 |
|  | Germany | 1 | 0 | 0 |
|  | Ghana | 1 | 100 | 1.82 |
|  | India | 3 | 66.67 | 3.64 |
|  | Iraq | 9 | 44.44 | 7.27 |
|  | Jordan | 2 | 50 | 1.82 |
|  | Kenya | 1 | 0 | 0 |
|  | Lebanon | 3 | 33.33 | 1.82 |
|  | Norway | 1 | 0 | 0 |
|  | Pakistan | 12 | 33.33 | 7.27 |
|  | Philippines | 1 | 0 | 0 |
|  | Sierra Leone | 1 | 0 | 0 |
|  | Sri Lanka | 10 | 60 | 10.91 |
|  | Turkey | 4 | 0 | 0 |
|  | Uganda | 5 | 60 | 5.45 |
|  | United Kingdom of Great Britain & Northern Ireland | 45 | 51.11 | 41.82 |
|  | USA | 9 | 22.22 | 3.64 |
|  | NA | 1 | 0 | 0 |

From table 4 we observe that the majority of Hub members are from the UK, Pakistan, and Sri Lanka while the majority of respondents were from the UK, reflecting the Hub demographics, however, only around half of the UK Hub members responded to the survey. A high number of respondents were from Sri Lanka and Colombia, with a high number of respondents from these groups. Table 4 also shows that the Hub members were spread across different streams, with the majority belonging to the Advisory Board, Migration and Displacement, and Transformation and Empowerment streams. The response rate was highest amongst the MICA and Livelihood, Land & Rights Streams. The majority of respondents were from the Migration and Displacement, and Transformation and Empowerment streams, reflective of the overall Hub stream patterns.

When conducting social network analysis, a low response rate can result in an incomplete network (or missing edge data). An incomplete network can cause analytical challenges, as many network metrics and statistics are defined for complete networks^[[5]](#footnote-5)^. In this setting, a non-respondent is defined as someone who does not complete the survey, and therefore does not provide any information on their out-going ties.

There are numerous approaches to handling missing network data, ranging from multiple imputation methods that draw on complex models (where a model is estimated and then used to probabilistically predict missing ties), to listwise deletion (the case where nodes who did not complete the survey are deleted form the network)^[[6]](#footnote-6)^. Model based approaches, especially Bayesian models, have been found to be particularly useful to impute missing tie information^[[7]](#footnote-7)^. However, model-based approaches also suffer from some disadvantages, more specifically, they are often difficult to implement (requiring complex model to be specified and estimated), and can result in introducing bias by imputing edges that over generalise the tendencies observed in other parts (i.e. information rich areas) of the network. The listwise strategy, the case of removing all nodes that did not respond to the survey^[[8]](#footnote-8)^, is the simplest approach to deal with missing data, and is a frequently used strategy observed in network studies^[[9]](#footnote-9)^.

In this study, we examine two networks; the first is the network of all individuals, including those that completed the survey and those that have been nominated but did not complete the survey, we refer to this as the full network. The second network is where a listwise operation has been implemented, only retaining the individuals (and edges amongst them) that completed the survey; we refer to this as the partial network.

Table 5: Characteristics of the full and partial networks

|  | Full Network | Partial Network |
| --- | --- | --- |
| Size | 121 | 55 |
| Density | 0.1598 | 0.3646 |
| Reciprocity | 0.1956 | 0.4192 |
| Betweenness Centralisation | 0.0634 | 0.1357 |
| Closeness Centralisation | 0.8038 | 0.5829 |
| Eigenvector Centralisation | 0.6963 | 0.5436 |
| Out Degree Centralisation | 0.8068 | 0.5798 |
| In Degree Centralisation | 0.2318 | 0.5057 |
| Clustering coefficient transitivity | 0.4679 | 0.6728 |
| Degree Assortativity | -0.229 | -0.2601 |

Figure 1: Plot of the number of respondents from each country**
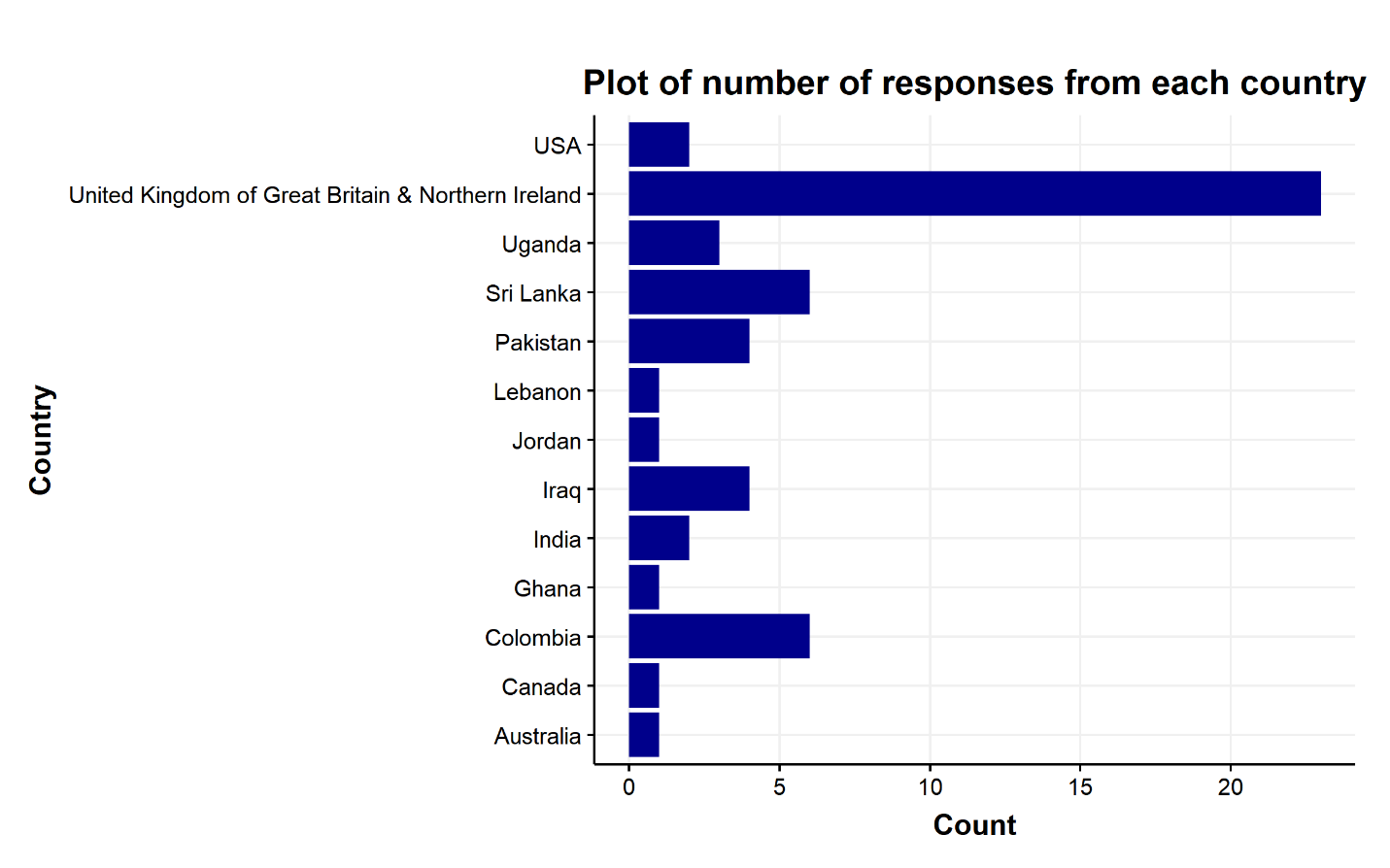
**

**Methods**

The network survey was developed online using the survey software Qualtrics. The survey was drafted by the core research team, piloted with five Hub members before it was refined and distributed. For each Hub member, participants answered whether they had a *current* connection with another individual and if they had one *before* they joined the Hub. Connections were rated on a scale, which allowed us to explore the relationships and the strength of these ties. If the connection had changed since joining the hub, participants described how this had happened (e.g., through a Hub convention, outside of work etc.). Finally participants were asked if they would like their professional connection to change by the end of the Hub. For ease of completion, we provided a short biography and a picture of each Hub member in the survey. Participants were provided with an overview of the study, its objectives and were asked to provide their informed consent. They also completed a short demographics section, where they provided for example, their age, gender, career stage and when they joined the Hub. This allowed us to capture if people connected more with other members similar to them (e.g. in terms of career stage or location).

**Results**

Once the survey was closed, survey data was downloaded from Qualtrics in csv format. Data was cleaned, processed and analysed in R and network visualisations were created using the Visone software. A number of network metrics were used to examine the structure of the network, including density, reciprocity, centralisation, degree assortativity and the E-I index.  Density captures the level of connectivity in the network, and reciprocity captures the proportion of ties that are two-way (or reciprocated). Centralisation measures capture the extent to which centrality is evenly distributed across actors in the network or concentrated in a small handful. For example, a network characterised with high degree centralisation would suggest that degree centrality (the number of connections an actor has in the network) is concentrated in a small number of actors (with a small number of actors having a large degree centrality, and most having a very small degree centrality), where the network structure would resemble a hierarchical star-like structure^[[10]](#footnote-10)^ Initial analysis of the network indicated that it was characterised by mild centralisation patterns. Degree assortativity captures the correlation between degree centrality of connected actors in a network^[[11]](#footnote-11)^ where scores can range between -1 and 1. A positive score suggests that actors with similar degree centrality are likely to connect. Whereas a negative score suggests that high degree actors are connected to many low degree actors, pointing towards a pattern of disassortativity in the network.

The analysis of this network pointed towards disassortativity (with consistent results observed for the full and partial networks), suggesting that high degree actors connected to many low degree actors in this case. This matches the expectation of how early stages of the Hub as a network would look like. The setting up of the Hub-level structure was run by a central Management Impact Communications Administration (MICA) team as well as by the Executive Group, consisting of 12 Co-Directors in charge of each research stream. This small group of people (high degree actors) have connections to almost every individual in the network.

The E-I index is a measure that captures whether ties are likely to occur internally (I) to a group or externally (E) to a group within a network^[[12]](#footnote-12)^. In the initial analysis, this was implemented for the stream group that actors belonged to. In this case the E-I index captured whether connections were more likely within streams or between streams. The results (for both the full and partial networks) indicate that ties tend to be external, that is between individuals belonging to different streams, rather than the same stream. The survey also asked participants if the connection had changed since joining the hub to describe how this had happened. The results indicated that the majority of the change was a result of Hub activity, more specifically “Through the Hub, not at a convention” or “At a Hub convention”.

Table 6: E-I Index results for the full network including the number of ties internal to a group and the number of ties external to the group.

Full network: 0.7027 (Network level E-I index)

| Group | External ties | Internal ties | Group E-I index |
| --- | --- | --- | --- |
| Advisory Board | 622 | 32 | 0.9021 |
| Law and Policy Frameworks | 228 | 6 | 0.9487 |
| Livelihood, Land & Rights Stream | 198 | 8 | 0.9223 |
| Masculinities and Sexualities | 398 | 18 | 0.9135 |
| Methodological Innovation | 344 | 8 | 0.9545 |
| MICA | 662 | 46 | 0.8701 |
| Migration and Displacement | 465 | 56 | 0.785 |
| Transformation and Empowerment | 981 | 171 | 0.7031 |
| NA | 54 | 0 | 1 |

Table 7: E-I Index results for the partial network including the number of ties internal to a group and the number of ties external to the group.

Partial network: 0.7211(Network level E-I index)

| Group | External ties | Internal ties | Group E-I index |
| --- | --- | --- | --- |
| Advisory Board | 309 | 19 | 0.8841 |
| Law and Policy Frameworks | 91 | 1 | 0.9783 |
| Livelihood, Land & Rights Stream | 100 | 4 | 0.9231 |
| Masculinities and Sexualities | 186 | 6 | 0.9375 |
| Methodological Innovation | 181 | 4 | 0.9568 |
| MICA | 399 | 42 | 0.8095 |
| Migration and Displacement | 155 | 11 | 0.8675 |
| Transformation and Empowerment | 443 | 64 | 0.7475 |

**Tomorrow’s Cities Full Technical Case Study**

**Study design and data**

Our data stems from two databases. One being an administrative database which contains generic information about collaborators in the Tomorrow’s City (TC) project such as their name, gender, organization, discipline they are trained in, their location or whether they are an early career scholar. We anonymized this information in the subsequent analysis. A second component then contains their connections with other collaborators in the network. To elicit such connections, we utilized SumApp, a platform which allows generating and managing networks. Hereby, we generated a list of all collaborators using the administrative data available. Collaborators where then able to click on the collaborator and indicate whether they collaborate closely, regularly, on occasion or just have been introduced. Other network members that were not selected by a respondent are thus assumed to have no collaborative connection. The data was collected February to March 2021.

We surveyed all individuals associated with the TC project including 174 individuals. In total, we obtained network information from 174 collaborators. Hereby, 47 percent of collaborators just show incoming ties which means they were mentioned by other collaborators but did not record any connections on their own. We assume that they thus have not participated in the network survey. The table shows comparison between the respondents and non-respondents.

Table 8 Characteristics of respondents versus full sample for data we can compare

| **Attribute** | **Corrected sample^[[13]](#footnote-13)^** | **Respondents** | **Non-respondents** |
| --- | --- | --- | --- |
| N | 174 | 92 | 83 |
| Female | 64 (36.7%) | 41 (44.5%) | 23 (28.0%) |
| ECR status | 77 (44.2%) | 47 (51.1%) | 30 (36.5%) |
| Location |  |  |  |
| Global | 24 (13.8%) | 20 (21.7%) | 4 (4.8%) |
| Multiple | 17 (9.8%) | 14 (15.2%) | 3 (3.6%) |
| Nairobi | 30 (17.2%) | 14 (15.2%) | 16 (19.5%) |
| Kathmandu | 39 (22.4%) | 16 (17.4%) | 23 (28.0%) |
| Quito | 22 (12.6%) | 16 (17.4%) | 6 (7.3%) |
| Istanbul | 28 (16.9%) | 12 (13.0%) | 16 (19.5%) |

Source: TC network data

To correct for such non-response bias, we use the reconstruction method discussed in Huang, Zhang, and Li (2019)^^[[14]](#footnote-14)^^. Hereby, we maximize the information present in our network and assume that incoming ties are reciprocal for non-respondents. Thus, each time they were mentioned by another collaborator, we assume that this connection is mutual and assign an outgoing tie. While we do not know whether this holds true in practice, we are able to maintain reported connections as dropping non-respondents from our sample would have deleted on average 14 connections per non-respondent.

## **Methods**

Upon completion of the survey, we downloaded the data from SumApp. In total, it includes 174 nodes (individuals) and 4706 edges (connections). The data was cleaned, processed and analysed in STATA and R whereby further analysis and network visualizations were created using Gephi. We use multiple network metrics to explore the structure of the TC network. This includes average degree, density, average path length, a clustering coefficient, and network diameter (see for example, Newman 2010)^[[15]](#footnote-15)^. Average degree describes the average amount of connections (edges) across individuals (nodes) in the network whereby the average path length displays the average number of edges along the shortest paths for all possible pairs of nodes. Along with density, which displays the percentage of realized out of all possible paths, these measures express connectivity within the network. This also includes network diameters, which express the shortest path between the two individuals who are furthest apart in the network. In the case of the TC network, a higher connectivity would imply higher levels of collaboration. A global clustering coefficient expresses the amount of closed triplets over the number of all triplets being open and closed ones^[[16]](#footnote-16)^. A triplet is a shared contact between two collaborators. Thus, the clustering coefficient expresses how likely it is that collaborators share a common contact and hence whether a network exhibits cliques.

**Results**

As mentioned, we are especially interested in aspects of cross-collaboration. Tables 9 - 11 present the results through partitioning the network by node attributes such as career level and organization, location and segment, as well as gender and discipline. This allowed us to see how collaboration differs depending on, for instance, where individuals are based. In addition, we compared mentioned network statistics for inter and intra edges. Inter edges only account for collaboration that occur across a certain criterion, e.g. across career level. Intra edges then only account for collaboration within the same criterion, e.g. within career level.

Table 9 Results of network metrics for collaboration across location

| Network metrics | General | Across all segments | Across non-UK segments | Within non-UK segments | Within UK segment |
| --- | --- | --- | --- | --- | --- |
| Nodes | 174 | 174 | 94 | 94 | 80 |
| Edges | 4704 | 2316 | 212 | 737 | 1651 |
| Average degree | 27.0 | 13.3 | 1.2 | 4.2 | 9.5 |
| Density | 0.16 | 0.08 | 0.01 | 0.02 | 0.16 |
| Average path length | 1.92 | 2.35 | 2.65 | 1.67 | 1.80 |
| Clustering coefficient | 0.43 | 0.14 | 0.20 | 0.59 | 0.55 |
| Diameter | 4 | 5 | 6 | 4 | 3 |

Source: TC network data

Table 10 Results of network metrics for cross-collaboration by location and career level

| Network metrics | All | | | Non-UK based | | | UK-based | | |
| --- | --- | --- | --- | --- | --- | --- | --- | --- | --- |
|  | ECR | Senior | Across | ECR | Senior | Across | ECR | Senior | Across |
| Nodes | 77 | 96 | 171 | 44 | 49 | 93 | 33 | 47 | 80 |
| Edges | 924 | 1664 | 2110 | 291 | 205 | 453 | 208 | 760 | 682 |
| Average degree | 12.0 | 17.33 | 12.3 | 6.6 | 4.2 | 4.9 | 6.3 | 16.2 | 8.5 |
| Density | 0.16 | 0.18 | 0.07 | 0.15 | 0.08 | 0.05 | 0.20 | 0.35 | 0.10 |
| Average path length | 1.96 | 1.88 | 2.5 | 2.3 | 2.73 | 3.07 | 1.99 | 1.69 | 2.38 |
| Clustering coefficient | 0.42 | 0.47 | . | 0.45 | 0.29 | . | 0.40 | 0.63 | . |
| Diameter | 3 | 4 | 5 | 5 | 6 | 8 | 4 | 3 | 5 |

Source: TC network data

Table 11 Results of network metrics for cross-collaboration by location and gender

| Network metrics | All | | | Non-UK based | | | UK-based | | |
| --- | --- | --- | --- | --- | --- | --- | --- | --- | --- |
|  | Women | Men | Across | Women | Men | Across | Women | Men | Across |
| Nodes | 64 | 85 | 150 | 36 | 43 | 93 | 28 | 42 | 80 |
| Edges | 790 | 1470 | 2002 | 192 | 242 | 399 | 229 | 581 | 683 |
| Average degree | 12.3 | 17.3 | 13.3 | 5.3 | 5.6 | 4.3 | 8.2 | 13.83 | 8.5 |
| Density | 0.20 | 0.21 | 0.09 | 0.15 | 0.13 | 0.05 | 0.30 | 0.34 | 0.02 |
| Average path length | 2.00 | 1.86 | 2.49 | 2.30 | 2.44 | 3.02 | 1.79 | 1.71 | 2.28 |
| Clustering coefficient | 0.53 | 0.45 | . | 0.44 | 0.39 | . | 0.65 | 0.57 | . |
| Diameter | 4 | 4 | 5 | 5 | 5 | 7 | 3 | 4 | 5 |

Source: TC network data

1. Humphries, M. D., & Gurney, K. (2008). Network ‘Small-World-Ness’: A Quantitative Method for Determining Canonical Network Equivalence. *PLoS ONE*, *3*(4), e0002051. https://doi.org/10.1371/journal.pone.0002051 [↑](#footnote-ref-1)
2. Kolaczyk, E. D., & Csárdi, G. (2014). *Statistical analysis of network data with R* (Vol. 65). New York: Springer. [↑](#footnote-ref-2)
3. Hobson, E. A., Silk, M. J., Fefferman, N. H., Larremore, D. B., Rombach, P., Shai, S., & Pinter‐Wollman, N. (2021). A guide to choosing and implementing reference models for social network analysis. *Biological Reviews*, *96*(6), 2716-2734. [↑](#footnote-ref-3)
4. Delabouglise, A., Antoine‐Moussiaux, N., Tatong, D., Chumkaeo, A., Binot, A., Fournié, G., ... & Peyre, M. (2017). Cultural practices shaping zoonotic diseases surveillance: the case of highly pathogenic avian influenza and Thailand native chicken farmers. *Transboundary and emerging diseases*, *64*(4), 1294-1305. [↑](#footnote-ref-4)
5. Borgatti, S. P., & Molina, J. L. (2003). Ethical and Strategic Issues in Organizational Social Network Analysis. *The Journal of Applied Behavioral Science*, *39*(3), 337–349. https://doi.org/10.1177/0021886303258111 [↑](#footnote-ref-5)
6. Newman, D. A. (2014). Missing Data: Five Practical Guidelines. *Organizational Research Methods*, *17*(4), 372–411. <https://doi.org/10.1177/1094428114548590>; Wang, C., Butts, C. T., Hipp, J. R., Jose, R., & Lakon, C. M. (2016). Multiple imputation for missing edge data: A predictive evaluation method with application to Add Health. *Social Networks*, *45*, 89–98. https://doi.org/10.1016/j.socnet.2015.12.003 [↑](#footnote-ref-6)
7. Krause, R. W., Huisman, M., Steglich, C., & Snijders, T. (2020). Missing data in cross-sectional networks – An extensive comparison of missing data treatment methods. *Social Networks*, *62*, 99–112. https://doi.org/10.1016/j.socnet.2020.02.004 [↑](#footnote-ref-7)
8. Pepinsky, T. B. (2018). A Note on Listwise Deletion versus Multiple Imputation. *Political Analysis*, *26*(4), 480–488. https://doi.org/10.1017/pan.2018.18 [↑](#footnote-ref-8)
9. Smith, J. A., & Moody, J. (2013). Structural effects of network sampling coverage I: Nodes missing at random. *Social Networks*, *35*(4), 652–668. https://doi.org/10.1016/j.socnet.2013.09.003 [↑](#footnote-ref-9)
10. Borgatti, S. P., & Molina, J. L. (2003). Ethical and Strategic Issues in Organizational Social Network Analysis. *The Journal of Applied Behavioral Science*, *39*(3), 337–349. https://doi.org/10.1177/0021886303258111 [↑](#footnote-ref-10)
11. Newman, D. A. (2014). Missing Data: Five Practical Guidelines. *Organizational Research Methods*, *17*(4), 372–411. https://doi.org/10.1177/1094428114548590 [↑](#footnote-ref-11)
12. Krackhardt, D., & Stern, R. N. (1988). Informal networks and organizational crises: An experimental simulation. *Social Psychology Quarterly, 123–140.* [↑](#footnote-ref-12)
13. [↑](#footnote-ref-13)
14. Huang, Feifei, Minqiang Zhang, and Yan Li. 2019. ‘A Comparison Study of Tie Non-Response Treatments in Social Networks Analysis’. *Frontiers in Psychology* 9: 2766. <https://doi.org/10.3389/fpsyg.2018.02766>. [↑](#footnote-ref-14)
15. Newman, Mark. 2010. *Networks: An Introduction*. Oxford, New York: Oxford University Press. [↑](#footnote-ref-15)
16. Wasserman, Stanley, and Katherine Faust. 1994. *Social Network Analysis: Methods and Applications*. Cambridge: Cambridge University Press. <http://ebooks.cambridge.org/ref/id/CBO9780511815478>. [↑](#footnote-ref-16)
